# Does cytomegalovirus infection increase the risk of tuberculosis in UK children?

**DOI:** 10.1098/rstb.2024.0457

**Published:** 2025-11-06

**Authors:** Sarah May Johnson, Elizabeth Whittaker, James Seddon, Beate Kampmann, Helen Payne

**Affiliations:** ^1^ Department of Paediatric Infectious Diseases, Imperial College London, London SW7 2AZ, UK; ^2^ Desmond Tutu TB Centre, Stellenbosch University, Stellenbosch 7505, South Africa; ^3^ Department of Paediatric Infectious Diseases and Immunology, Imperial College London, London SW7 2AZ, UK; ^4^ The Vaccine Centre, London School of Hygiene and Tropical Medicine, London WC1E 7HT, UK

**Keywords:** tuberculosis, cytomegalovirus, TB exposure, children

## Abstract

There is a hypothesized association between pre-existing cytomegalovirus (CMV) infection and risk of acquiring tuberculosis (TB). We aimed to explore if CMV seroprevalence and CMV IgG levels in children were associated with TB disease or *Mycobacterium tuberculosis* (*Mtb*) infection compared with children who were exposed to TB but remained well. In this cross-sectional analysis from an observational cohort study of children exposed to TB in their household in the UK, we examined samples from 75 participants, of whom 40 (53%) were male. Median age of the cohort was 6 years (interquartile range: 3–11 years). In total, 21 (28%) children had TB disease, 27 (36%) had *Mtb* infection and 27 (36%) had TB exposure only. There was no increased risk of TB in children who were CMV-seropositive (OR 2.18 [0.75–6.48]), and there were no differences in CMV IgG quantification by TB category. There was no detectable CMV viraemia in any of the children in our study. We found higher levels of CMV seroprevalence (49%) than previously described in the UK. In this small study of children exposed to TB, in a low-TB burden setting, we found no association between CMV serostatus or CMV IgG levels and TB status.

This article is part of the discussion meeting issue ‘The indirect effects of cytomegalovirus infection: mechanisms and consequences’.

## 1. Background

One million children globally are estimated to develop tuberculosis (TB) disease each year, and approximately one-quarter of these children die from TB [1,2]. Despite a wealth of research into TB pathogenicity, it is not fully understood why some children progress to TB disease. Recent research suggests cytomegalovirus (CMV) infection may play a key role in the progression from *Mycobacterium tuberculosis* (*Mtb*) infection to TB disease [3–9].

In England, CMV seropositivity has been reported as 10–34% in the first 2 years of life [10–12], rising to 23% in adolescents [12]. The proportion seropositive in London adolescents and young adults (11−24-year-olds) was found to be higher, at 50% [12]. In adults, this proportion has been found to reach 70% in European women of child-bearing age [13]. Despite being a ubiquitous virus globally, CMV has not been extensively studied [5]. However, there is growing interest in the long-term and indirect effects of CMV, causing inflammation and being associated with cancer risk and cardiovascular disease in immunocompetent adults [14] and atopy in children [10].

The epidemiology of individuals with CMV and TB has considerable overlap [4,6,15]. For both TB and CMV, humans are the primary host and similar risk factors predispose to infection, and both TB and CMV can remain latent for years before re-activation [4]. CMV leads to immune activation and dysregulation [4,16], and it is suggested that CMV impairs the immunological response to *Mtb* infection. Infants with CMV infection in the first year of life were at greater risk of developing TB disease between the age of 1 and 9 years in a prospective birth cohort in South Africa [17]. Notably, Martinez *et al.* [17] found that those with the highest CMV viral load had the highest risk of developing future TB. In addition, TB disease has been associated with higher CMV IgG levels [18].

We aimed to describe CMV seroprevalence and CMV IgG levels in children with TB disease, *Mtb* infection and TB exposure. In addition, we sought to explore if children with TB disease were more likely to have a current CMV infection or evidence of previous CMV infection.

## 2. Methods

### (a) Participants

Participants aged 0−16 years were recruited between 1 January 2011 and 31 December 2014 from 11 paediatric TB clinics in the UK, as part of the NIKS Cohort Study. In 2006, the National Institute for Health and Care Excellence (NICE) in the UK recommended that following household TB exposure, children should be evaluated using tuberculin skin testing (TST). Those with positive TST results should undergo interferon-gamma release assay (IGRA) evaluation and only those with positive results would be eligible for TB preventive treatment. The NIKS Cohort Study sought to evaluate the negative predictive performance of IGRA in TST-positive children. Serial serum samples were taken at baseline and over the period of follow-up [19]. Children who had previously had a positive test for TB were excluded. Of the 392 participants of the NIKS Cohort Study, samples were only available from a sub-set of 75 children from six of the study sites (The Royal London, Birmingham, Bristol, St Mary’s, Northwick Park and Southampton hospitals). Data available included age, sex, TB symptoms at first clinic appointment, TB diagnosis, TB treatment, Bacillus Calmette-Guérin (BCG) status and TST result, and IGRA results.

### (b) Study design

In this cross-sectional NIKS sub-study, samples were obtained from three clinical groups: children with TB disease, those with *Mtb* infection and those with TB exposure (household TB contact, but no evidence of *Mtb* sensitization). Evaluation for TB disease in the original NIKS Cohort Study for TB disease included history, examination, chest radiography, TST and IGRA tests, and microbiology if indicated. TB disease was diagnosed based on NICE guidelines for children above the age of 2 years, in conjunction with the clinical context, by study paediatricians [19].

*Mtb* infection included children with a positive TST and/or a positive IGRA. A positive TST was defined as a transverse diameter of the induration ≥6  mm in BCG-unvaccinated children and ≥15  mm in vaccinated children, in line with the 2006 NICE recommendations. The child was classified as IGRA-positive if either the baseline or the 2-month IGRA was positive. Further details are available in the NIKS Cohort Study [19].

Healthy children who were household TB contacts and had no evidence of *Mtb* sensitization were classified as having no evidence of TB disease or *Mtb* infection.

### (c) Samples

Serum samples were obtained from the participants’ first clinic visit. Samples were stored at −80°C. Samples of < 250 µl were excluded owing to insufficient sample remaining for both polymerase chain reaction (PCR) and enzyme-linked immunosorbent assay (ELISA) to be carried out.

### (d) Laboratory analyses

CMV-specific immunoglobulin G (IgG) was measured using GenWay Biotech Cytomegalovirus IgG ELISA as per manufacturer’s instructions, and optical density was used to estimate the quantity of CMV-specific IgG using the VersaMax ELISA Microplate reader. Seropositivity was defined as a CMV IgG detected at >1.1 IU ml^−1^. The QIAGEN QIAamp^®^ Blood kit was used to extract DNA from serum, and CMV DNA was quantified by real-time PCR using the bioMérieux ARGENE CMV R-GENE^®^ kit. Samples were run in triplicate on an Applied Biosystems StepOnePlus Real-time PCR system machine. Maximum sensitivity of the assay was 446 copies ml^−1^. Presence of CMV IgG in serum was taken to represent anytime exposure to CMV, and presence of CMV DNA in serum was defined as acute infection.

### (e) Statistical analysis

Stata^TM^ 16.1 was used for all analyses. A Kruskal–Wallis equality-of-populations rank test was carried out for CMV IgG level, and evaluated children with TB disease, *Mtb* infection and TB exposure as three independent groups. A second Kruskal–Wallis equality-of-populations rank test analysed the relationship between CMV IgG level and age. An odds ratio was calculated to determine association between CMV seropositivity and TB clinical state.

### (f) Ethics

The original NIKS cohort study had ethical approval from the UK National Research Ethics Service (REC: 11/11/11). Parents of all children included in the study provided written informed consent, with additional assent from older children [19].

## 3. Results

### (a) Patient characteristics

Seventy-five participants were included, of whom 40 (53%) were male, and the median age was 6 years (interquartile range (IQR): 3−11 years; table 1). In total, 21 (28%) had TB disease, 27 (36%) had *Mtb* infection and 27 (36%) had TB exposure without evidence of TB disease or *Mtb* infection.

**Table 1.** Demographics of participants and by tuberculosis (TB) diagnosis. *Mtb*, *Mycobacterium tuberculosis*.

*age* (years)	participants 75	TB disease 21 (28%)	*Mtb* infection 27 (36%)	no evidence of TB disease or *Mtb* infection 27 (36%)
	*N* (%)	*N*	*N*	*N*
0−2	18 (24)	5	4	9
3−5	15 (20)	4	5	6
6−10	20 (27)	5	7	8
11+	22 (29)	7	11	4
**sex**				
male	40 (55)	11	15	14
female	35 (45)	10	12	13
*BCG scar*				
present	29 (38)	8	12	9
not present	46 (61)	13	15	18

### (b) Cytomagalovirus viral load

None of the participants was found to have active CMV infection as defined by CMV DNA detected by DNA PCR from serum. None of the samples amplified above the lowest level of detection of the assay.

### (c) Cytomagalovirus seropositivity and IgG levels

**Figure 1.**
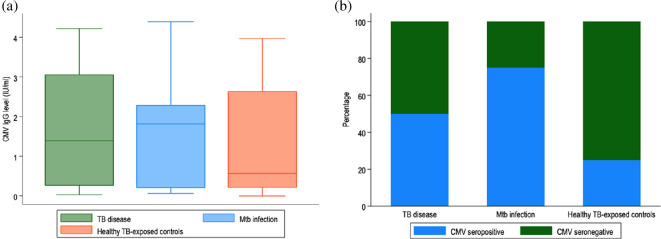
(a) Boxplot of cytomegalovirus (CMV) IgG level by tuberculosis (TB) clinical state, in those who were seropositive. CMV IgG level was measured in IU ml^−1^, with >1.1 IU ml^−1^ considered as seropositive. (b) Percentage of CMV seropositivity by TB clinical state. Mtb, *Mycobacterium tuberculosis*.

The median CMV IgG level was 1.52 IU ml^−1^ (IQR: 0.22−2.75). A Kruskal–Wallis equality-of-populations rank test of CMV IgG level by TB clinical state (TB disease, *Mtb* infection, exposed to TB) was not significant; *χ*^2^ with ties = 2.4 with 2 d.f. (*p* = 0.3; figure 1a).

Seropositivity increased with age (table 2), and about half of patients were CMV IgG positive. A Kruskal–Wallis equality-of-populations rank test of CMV IgG level by age group was found not to be statistically significant (*χ*^2^ with ties = 2.9 with 3 d.f. (*p* = 0.4)). Figure 1b shows the percentage of CMV seropositive patients by TB group. The odds ratio measuring the association between CMV seropositivity and risk of having either TB disease or *Mtb* infection was 2.18 (95% CI: 0.75−6.48, *χ*^2^ = 2.55, *p* = 0.1).

**Table 2.** Cytomegalovirus (CMV) seroprevalence and mean antibody level by age group.

	CMV serology		
age (years)	negative	positive (>1.1 IU ml^−1^)	mean antibody level (IU ml^−1^) [95% CI]
0−2	11 (61%)	7 (39%)	1.36 [0.67−2]
2−5	8 (53%)	7 (47%)	1.38 [0.63−2.13]
6−10	11 (55%)	9 (45%)	1.37 [0.81−1.93]
11+	8 (36%)	14 (64%)	2.16 [1.62−2.68]
all ages	38 (51%)	37 (49%)	1.68 [1.39−1.98]]

## 4. Discussion

Our results showed no association between TB clinical state and CMV seropositivity and no association between the level of CMV-specific IgG and TB clinical state. Our findings are not consistent with previous studies showing that CMV and TB were associated [3–8,20] and there are many potential explanations for this. The first is that our sample size may be too small to detect real differences. Second, given that this study was conducted in a low-TB burden setting, this may explain the difference between our results and the previous studies conducted in high-TB burden settings. In addition, the differences in study design (household exposure to TB) could have introduced biases relating to the high proportion of CMV IgG seropositivity.

We describe higher rates of CMV seroprevalence than previously reported in the UK. Overall, half of the patients were CMV IgG positive. This sub-study cohort had higher rates of CMV seroprevalence than reported in the UK [10–12] and in Germany (21% in 1−2 year olds and 32% in 14−17 year olds) [21]. It could be that CMV seroprevalence is increasing in the UK. Given that this group of children were exposed to TB in their households, it is also likely that they are a vulnerable population, and there may be overlapping risk factors that similarly exposed these children to CMV.

None of the children from our study demonstrated active CMV infection as defined by detectable CMV viraemia in serum samples. A limitation that may in part account for this is the use of frozen serum for CMV DNA detection. Whole-blood analysis, which includes CMV incorporated into white cells in the blood, would be a more sensitive test for viraemia [22,23], but was not available.

Our findings are from a low-TB burden setting and may not be generalizable to other settings (e.g. to acutely unwell patients or to high-TB burden settings), where there may be differences in immune activation seen [24]. A further limitation is that we did not have access to other demographic factors, including ethnicity, family size and HIV infection status. The proportion of children living with HIV in the UK is, however, very low [25,26]. Furthermore, we were unable to analyse the full NIKS cohort owing to sample shortages, and this opportunistic sampling may not be representative of the whole cohort. We also acknowledge that our cohort of TB-exposed children is not necessarily representative of the general population.

In this low-TB setting, we did not demonstrate a relationship between CMV and TB clinical state in children. Future research should include longitudinal cohort studies to determine the incidence of TB clinical states, including *Mtb* infection and TB disease developing in those exposed to TB, with and without CMV infection, and study the immunological responses of these participants over time.

## Data Availability

Data are available at https://doi.org/10.5061/dryad.gxd2547wv.
